# Antibiotics should not be used for back/leg pain

**DOI:** 10.1080/17453674.2020.1855561

**Published:** 2020-12-11

**Authors:** Peter Fritzell, Tomas Bergström, Bodil Jönsson, Siv G E Andersson, Mikael Skorpil, Peter Muhareb Udby, Mikkel Andersen, Olle Hägg

**Affiliations:** a RKC Centre for Spine Surgery in Stockholm, Sweden/Futurum, Academy for Health and Care, Region Jönköping County , Sweden ;; b Department of Infectious Diseases, Institute of Biomedicine, University of Gothenburg , Sweden ;; c Sahlgrenska University Hospital , Göteborg , Sweden ;; d Department of Cell and Molecular Biology, Biomedical Center, Uppsala University , Sweden ;; e Karolinska University Hospital , Stockholm , Sweden ;; f Spine Unit, Ortopaedkirurgisk Afdeling, Sjaellands Universitetshospital , Køge , Denmark ;; g Spine Center of Southern Denmark, Lillebaelt Hospital , Middelfart , Denmark ;; h Spine Center Göteborg , Västra Frölunda , Sweden

Antibiotics have been suggested as treatment for selected patients with chronic back pain, with or without leg pain, and in association with Modic changes type 1 (MC1) on MRI (Modic et al. 1988, Albert et al. 2013a).

The hypothesis is that various bacteria, but above all the common skin bacterium Cutibacterium acnes (formerly Propionibacterium acnes), would spread hematogenously to degenerated discs, where a “low-grade subclinical infection” would trigger an inflammatory reaction and cause MC1 (Albert et al. 2013a), irritate nociceptive nerve endings, and induce pain. Other studies have not found this connection, or have been cautious in their conclusions (Birkenmaier 2013, Urquhart et al. 2015) but have not had the same public impact.

Since back/leg pain is common, and Modic changes occur in people with or without back/leg pain (Wang et al. 2012), and as antibiotic resistance is a major health threat (Carlsson et al. 2019), the suggestion of treating chronic back/leg pain with antibiotics must be thoroughly investigated.

Research groups in Sweden, Denmark, and Norway have independently conducted studies from 3 different perspectives, but with a focus on the same basic questions: is there a causative link between Modic changes, back pain, and bacteria. The studies, all published during 2019, conclude that antibiotics should not be used for back/leg pain, unless there is a clinically relevant infection in the disc/vertebra (discitis/spondylitis).

## Bacteria and discs, Fritzell et al. (2019)

**Figure 1.**
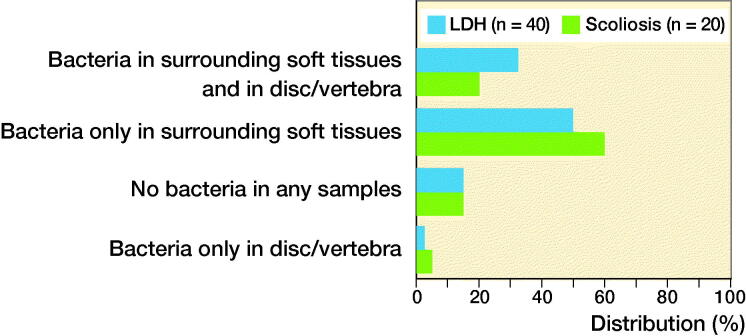
Data from Fritzell et al. 2019. LDH = lumbar disc herniation.

In this Swedish multicenter study, the presence of bacteria in discs/vertebrae was evaluated in 2 patient groups from 7 hospitals (Figure 1). Samples from degenerated discs in 40 patients operated on for lumbar disc herniation (median age 43 years) were compared with samples from non-degenerated discs in young patients operated on for scoliosis, who did not have back pain or disc herniation (median age 17 years). The samples were analyzed using culture and DNA technology at 2 independent university laboratories in Gothenburg and Uppsala.

The study found no statistically significant difference in bacterial presence between the 2 groups. No association was seen between Modic changes and findings of bacteria or between back/leg pain and findings of bacteria or Modic changes.

## Conclusion

Findings of bacteria, or traces of bacteria, as described in previous studies, are most likely a result of contamination during the surgical procedure.

### Modic changes and back pain, Udby et al. (2019)

**Figure 2.**
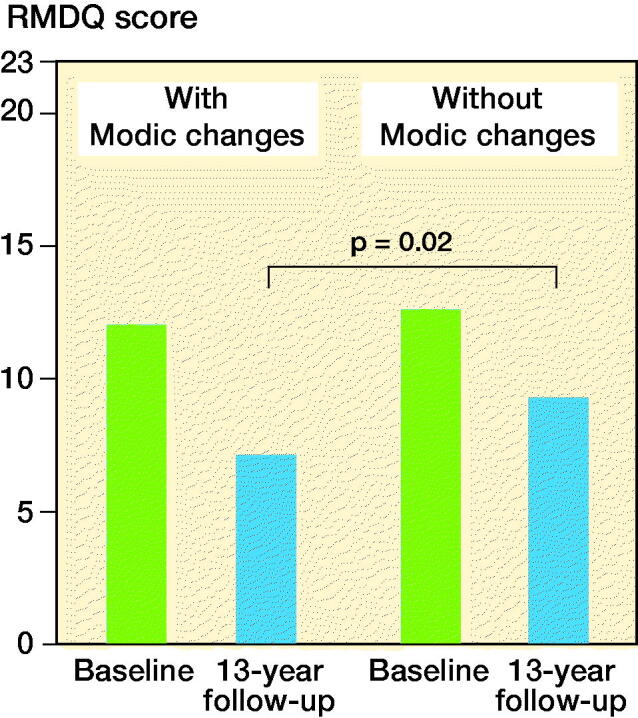
Data from Udby et al. 2019.

In Denmark, the clinical relevance of Modic changes for back pain and function was evaluated in a cohort study with a 13-year follow-up period (Figure 2). In all, 204 patients were stratified to a group with or without Modic changes. The relationship between Modic changes at study start and impaired physical function, measured with the RMDQ (Roland Morris Disability Questionnaire, 0 = best, 23 = worst possible function) (Roland et al. 1983), back pain, and sick leave at follow-up was evaluated.

The study found no statistically significant differences in demographic data, BMI, smoking, back and leg pain at the start of the study, or at follow-up. Physical function measured with RMDQ was initially equal between the 2 groups, but statistically significantly better (without clinical relevance) at the follow-up in patients with Modic changes at the start of the study, 7.4 versus 9.6 (p = 0.02). Patients with Modic changes at the start of the study also had fewer sick days due to back pain during the study period, 9 versus 23.

## Conclusion

Modic changes on MRI in patients with back pain at study start were not negatively associated with back pain or functional level after 13 years.

### Antibiotics and back pain, Bråten et al. (2019)

**Figure 3.**
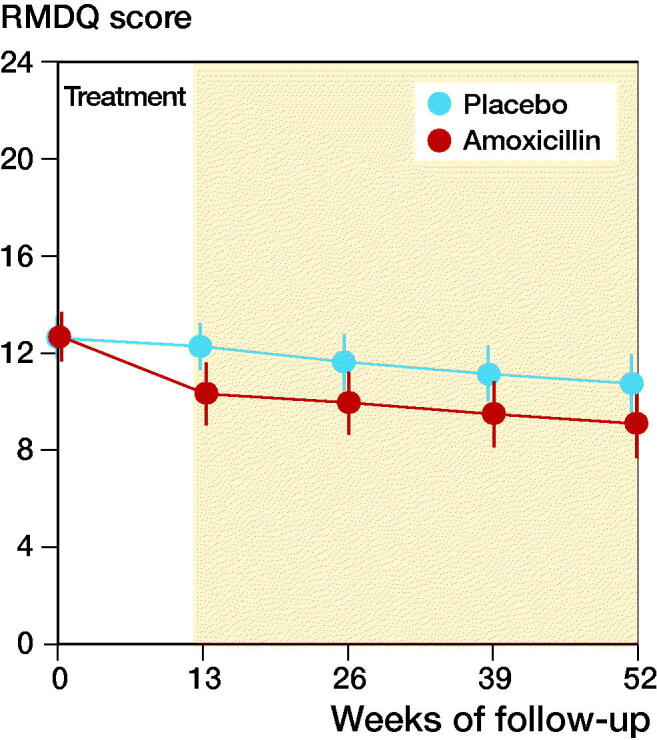
Data from Brеten et al. 2019.

In Norway, the Danish study by Albert et al. (2013b) was largely reproduced to evaluate the effect of antibiotic treatment in patients with chronic back pain (Figure 3). The research group conducted a randomized placebo-controlled double-blind multicenter study at 6 hospitals (the Albert et al. study was a single-center study). The Norwegian study included 180 patients with back pain and Modic change type 1 or type 2. The participants were randomized to 3 months of treatment with either amoxicillin 3 × 750 mg or placebo, and follow-up was done according to “intention to treat” after 1 year. The outcome, measured with RMDQ, was similar in the 2 groups.

## Conclusion

3 months of treatment with amoxicillin showed no relevant clinical effect after 1 year in patients with chronic back pain and Modic changes.

### Summary

Based on current scientific evidence, where the 3 Nordic studies supply complementary findings, antibiotic treatment for back/leg pain with no signs of serious infection (discitis/spondylitis) cannot be recommended. It is important to counteract the development of antibiotic resistance in society due to antibiotic use without scientific evidence.

This article has also been published in the Swedish Läkartidningen; 2020; 117: 20067.
